# Auxiliary Teaching and Student Evaluation Methods Based on Facial Expression Recognition in Medical Education

**DOI:** 10.2196/72838

**Published:** 2025-05-22

**Authors:** Xueling Zhu, Roben A Juanatas

**Affiliations:** 1 College of Computing and Information Technologies, National University, 551 Mariano Fortunato Jhocson Street, Sampaloc, Manila, 1008, Philippines, 86 13966689261

**Keywords:** medical education, facial expression recognition, auxiliary teaching, student assessment, teaching innovation

## Abstract

Traditional medical education encounters several challenges. The introduction of advanced facial expression recognition technology offers a new approach to address these issues. The aim of the study is to propose a medical education–assisted teaching and student evaluation method based on facial expression recognition technology. This method consists of 4 key steps. In data collection, multiangle high-definition cameras record students’ facial expressions to ensure data comprehensiveness and accuracy. Facial expression recognition uses computer vision and deep learning algorithms to identify students’ emotional states. The result analysis stage organizes and statistically analyzes the recognized emotional data to provide teachers with students’ learning status feedback. In the teaching feedback stage, teaching strategies are adjusted according to the analysis results. Although this method faces challenges such as technical accuracy, device dependency, and privacy protection, it has the potential to improve teaching effectiveness, optimize personalized learning, and promote teacher-student interaction. The application prospects of this method in medical education are broad, and it is expected to significantly enhance teaching quality and students’ learning experience.

## Introduction

Under the traditional mode of medical education, although rich teaching experience and knowledge system have been accumulated for a long time, some inherent problems have gradually emerged with the advancement of technology and changes in social needs [1,2]. First, in terms of teaching methods, traditional medical education generally adopts a teacher-led teaching mode, which often neglects students’ initiative and participation, and limits the cultivation of their independent thinking and practical abilities [3]. Second, in terms of evaluation mechanism, traditional medical education mainly relies on written examinations and clinical internships, but this evaluation method often cannot fully and accurately reflect students’ learning outcomes and abilities. Especially in nonacademic evaluations, such as emotions, attitudes, and values, traditional methods seem inadequate [4]. In addition, with the continuous updating of medical knowledge and the rapid development of technology, traditional medical education often lags behind the latest scientific research achievements and practical experience in teaching content and methods, which makes it difficult for students to adapt to the rapidly changing medical environment after graduation. Therefore, it is necessary to introduce new teaching methods and technological means to make up for the shortcomings of traditional medical education and improve the quality and effectiveness of medical education.

Facial expression recognition technology is a technique that recognizes and understands an individual’s emotional state by capturing and analyzing facial expressions [5-8]. The development history of facial expression recognition technology can be traced back to the 1970s when American psychologists Ekman and Friesen pioneered the definition of 6 basic human expressions and established the Facial Action Coding System, laying the foundation for subsequent research on facial expression recognition [9-11]. This milestone work not only clarifies the framework for facial expression classification but also provides standardized analytical tools for subsequent research [12].

Entering the 21st century, with the vigorous development of computer vision and artificial intelligence technology, facial expression recognition technology has ushered in new opportunities for development. In early research, researchers mainly relied on traditional image processing and machine learning algorithms such as support vector machines (SVMs) and principal component analysis to process and analyze facial expressions [13,14]. However, the recognition accuracy of these methods is limited by manually designed feature extraction methods and limited training data [15-17].

In recent years, the rise of deep learning technology has brought revolutionary changes to the field of facial expression recognition. Deep learning models such as convolutional neural networks (CNNs) and recurrent neural networks have achieved significant results in facial expression recognition [18-20]. For example, AlexNet proposed by Krizhevsky et al has made breakthrough progress in ImageNet image classification tasks, providing strong support for the application of deep learning in expression recognition [21]. Subsequently, researchers began to apply deep learning models to facial expression recognition tasks, continuously improving the accuracy and robustness of facial expression recognition by constructing deeper network structures and introducing attention mechanisms and other methods.

In specific research work, there are several aspects that deserve special attention. First, multimodal fusion technology has become a research hotspot in recent years. Researchers have found that in addition to facial expressions, a person’s emotional state can also be expressed through other modalities such as speech, posture, and physiological signals. Therefore, integrating information from multiple modalities can further improve the accuracy and reliability of facial expression recognition. For example, some studies have achieved more accurate emotion recognition by integrating facial expressions and voice information [22].

Second, the application of self-supervised learning in facial expression recognition is gradually receiving attention. Due to the high cost of obtaining labeled data, researchers have begun exploring the use of unlabeled data for pretraining to improve the model’s generalization ability. This self-supervised learning method can fully use a large amount of unlabeled data resources and extract useful feature representations by designing appropriate pretraining tasks. For example, some studies have achieved significant performance improvements by designing self-supervised learning tasks based on facial movements to pretrain models [23].

In addition, with the continuous development of computer vision technology, some new technologies have also been introduced into facial expression recognition. For example, 3D facial expression recognition technology can more accurately recognize facial expressions by capturing 3D information of the face. This technology can overcome issues such as lighting and posture in 2D images and improve the robustness of facial expression recognition. In addition, some researchers have also explored expression synthesis techniques based on generative adversarial networks, which can generate facial images with specific expressions, providing more training data and testing samples for expression recognition [24-26].

Introducing facial expression recognition technology to assist teaching and student assessment in medical education can bring significant advantages [27]. First, this technology can capture students’ facial expressions in real time, providing teachers with intuitive and immediate feedback, thereby helping them more accurately grasp students’ learning status, adjust teaching strategies in a timely manner, and improve teaching effectiveness. Second, facial expression recognition technology can help students better understand teaching content. Through students’ facial feedback, teachers can promptly answer students’ questions and enhance students’ learning motivation and participation [28]. In addition, facial expression recognition technology can also help achieve personalized teaching. Based on the emotional reactions of different students, teachers can develop more targeted teaching plans to meet their personalized needs [29].

Facial expression recognition technology has unique advantages in solving traditional medical education problems. Traditional medical education often relies on written examinations and clinical internships to evaluate students’ learning status, but this evaluation method is difficult to comprehensively and accurately reflect students’ learning outcomes and abilities. Especially for nonacademic evaluations of students’ emotions, attitudes, and values, traditional methods seem inadequate. Facial expression recognition technology can capture students’ emotional reactions, providing teachers with a more comprehensive evaluation basis, thereby more accurately assessing students’ comprehensive qualities and potential. In addition, traditional medical education often adopts a teacher-led teaching mode in teaching methods, lacking student participation and interaction. Facial expression recognition technology can obtain real-time feedback from students, making teaching more interactive and targeted and improving students’ learning outcomes.

## Facial Expression Recognition Technology and Its Application in Medical Education

### Principles of Facial Expression Recognition Technology

Facial expression recognition technology is a technique that uses image processing and analysis to recognize and understand human facial expressions. It is mainly based on algorithms, such as machine learning and deep learning, and achieves automatic recognition and classification of facial expressions through training and learning on a large number of facial expression images [30-33]. The facial expression recognition technology mainly relies on computer vision and pattern recognition technology and achieves automatic recognition and classification of facial expressions through feature extraction and classification of facial images [34]. This process can be summarized as the following steps:

- Image input: first, an image containing facial expressions needs to be input. These images can come from various sources, such as real-time video streams captured by cameras and static image libraries.
- Facial detection: using facial detection algorithms to accurately locate facial regions in the input image. This is one of the key steps in facial expression recognition technology, as only by accurately detecting the face can facial expression features be further extracted.
- Image preprocessing: preprocessing the detected facial images, including grayscale, normalization, denoising, and other operations. These preprocessing steps help eliminate redundant information and interference factors in the image, improving the accuracy of subsequent feature extraction.
- Feature extraction: in the preprocessed image, use feature extraction algorithms to extract key features that can characterize facial expressions. These features can include geometric features of the face (such as the shape and position of the eyes and mouth), texture features (such as skin texture and wrinkles), and dynamic features (such as the movement of facial muscles). Feature extraction is one of the core steps in facial expression recognition technology, and the quality of the extracted features directly affects the accuracy of subsequent facial expression recognition.
- Pattern classification: after extracting facial expression features, machine learning or deep learning algorithms are used to classify these features. These algorithms can automatically recognize different facial expression categories by training and learning from a large number of labeled facial expression images. Common machine learning algorithms include SVM, decision tree, and random forest; deep learning algorithms include CNN, recurrent neural networks, and so on. These algorithms continuously optimize model parameters during the training process to improve the accuracy and generalization ability of facial expression recognition.

### Potential Applications of Facial Expression Recognition in Medical Education

In medical education, facial expression recognition technology can be applied in 2 aspects: assisting teaching and student assessment [35]. First, teachers can analyze students’ facial expressions to understand their understanding and mastery of the teaching content and adjust teaching strategies and methods accordingly. Second, student assessment can also achieve more objective and accurate evaluation results through facial expression recognition technology. For example, students’ participation, interest, and learning status can be evaluated by analyzing their facial expressions during classroom discussions, experimental operations, and other processes.

### Auxiliary Teaching: Optimizing Teaching Strategies Through Facial Expression Recognition

In medical education, teachers can observe students’ facial expressions to understand their understanding and mastery of the teaching content [36]. Facial expression recognition technology can provide effective support for this. By analyzing students’ facial expressions in real time, teachers can quickly identify their emotional states such as confusion, interest, or fatigue. Based on this information, teachers can adjust teaching strategies and methods in a timely manner to better meet students’ learning needs.

Specifically, when teachers find that students are confused about a certain knowledge point, they can pause the explanation and clarify it through questioning or giving examples; when students show a strong interest, they can further expand their relevant knowledge points to stimulate their learning interest; and when students show fatigue, they can adjust the teaching pace appropriately and relieve their fatigue through rest or small games.

### Student Assessment: Achieving Objective and Accurate Assessment Through Facial Expression Recognition

In addition to assisting teaching, facial expression recognition technology can also be used for student assessment. Traditional student assessment methods often rely on students’ self-reports or teachers’ subjective judgments, which have certain subjectivity and errors. Facial expression recognition technology can evaluate students’ participation, interest, and learning status by analyzing their facial expressions, providing more objective and accurate evaluation results.

For example, in classroom discussions, teachers can evaluate students’ engagement by analyzing their facial expressions. When students actively participate in discussions, they usually show a positive and focused expression; when student engagement is low, they may exhibit expressions of boredom, fatigue, or confusion. By collecting and analyzing these facial expression data, teachers can more accurately evaluate students’ classroom performance.

In addition, facial expression recognition technology can also be used to evaluate students’ learning status in experimental operations. When students encounter difficulties or challenges, they usually show expressions of tension, anxiety, or confusion; when students successfully solve problems, they will show excited and satisfied expressions. By analyzing these facial expression data, teachers can understand students’ learning status during experimental operations and provide timely guidance and assistance.

## Auxiliary Teaching and Student Evaluation Methods Based on Facial Expression Recognition in Medical Education

### Overview of Methods

The auxiliary teaching and student evaluation method based on facial expression recognition in medical education proposed in this section mainly include the following 4 steps: data collection, facial expression recognition, result analysis, and teaching feedback.

### Data Collection

In the medical education–assisted teaching and student evaluation method based on facial expression recognition, the data collection process is the foundation and key of the entire process. The primary task of data collection is to obtain high-quality video and image data, which mainly comes from the facial expression records of students during the teaching process. To ensure the comprehensiveness and accuracy of data, multiple high-definition cameras are usually installed in the classroom to capture students’ facial expressions from different angles. These cameras need to have good resolution and frame rate to ensure clear and detailed images. In addition, portable devices such as smartphones and tablets can be used for supplementary shooting to meet the flexible data collection needs in special situations.

The collected data should not only include static facial images but also dynamic video recordings. This is because the changes in facial expressions are often continuous, and a single static image may not fully reflect the emotional state of students. Therefore, the collection of video data is crucial, as it can capture the changes in students’ expressions at different time points, providing richer information for subsequent expression recognition and analysis. At the same time, video data can also record classroom interactions, including scenarios such as students’ questions, answers, and discussions. These behavioral data also have important reference value for evaluating teaching effectiveness.

During the data collection process, special attention should be paid to privacy protection and data security. Due to the personal privacy involved in the collected facial expression data, it is necessary to strictly comply with relevant ethical norms and laws and regulations. Prior to data collection, informed consent from students should be obtained, clearly informing them of the purpose, scope of use, and protective measures of the data collection. At the same time, encryption technology is used in the process of data storage and transmission to prevent data leakage and ensure that student privacy is not violated. In addition, in order to further improve the confidentiality of the data, anonymization can be performed on the collected data, that is, personal identity information can be separated from facial expression data through technical means, thereby ensuring the effectiveness of data analysis while maximizing the protection of student privacy.

Finally, in order to ensure the high quality and practicality of the data, detailed standards and specifications need to be developed in the data collection process. These standards include the installation position and angle of the camera, control of lighting conditions, and selection of shooting time. For example, cameras should be installed at angles that can clearly capture students’ faces and avoid adverse lighting conditions such as backlighting. The shooting time should cover the entire teaching process, including explanations, interactions, and breaks, to comprehensively reflect the changes in students’ expressions at different stages of teaching. By formulating and implementing these standards and specifications, the accuracy and reliability of data collection can be maximized, providing a solid foundation for subsequent facial expression recognition and result analysis.

### Facial Expression Recognition

In medical education–assisted teaching and student evaluation methods based on facial expression recognition, the facial expression recognition process is a key step in converting the collected raw data into meaningful emotional information. The process of facial expression recognition first relies on advanced computer vision and machine learning technologies, which can automatically detect and analyze key feature points of students’ faces. Specifically, facial detection algorithms locate facial regions by processing video frames or images and further extract key feature points including eyes, eyebrows, nose, and mouth. The precise localization of these feature points is the foundation for facial expression recognition, ensuring that the algorithm can accurately capture subtle changes in the face.

After extracting key feature points, the expression recognition system will use the trained classifier to recognize different expression categories. Common classifiers include SVM and CNN. These classifiers can learn and distinguish feature patterns corresponding to various expressions by training on a large amount of labeled data. Especially, CNNs have shown superior performance in facial expression recognition, as they can automatically extract high-level feature representations, significantly improving the accuracy and robustness of facial expression recognition. In the recognition process, the system not only needs to distinguish basic expressions, such as happiness, sadness, and surprise, but also needs to combine contextual information to recognize complex emotional states, such as confusion, focus, or boredom.

To further improve the accuracy of facial expression recognition, multimodal fusion is usually required, which combines multiple sources of information such as facial expressions, speech emotions, and posture changes. Multimodal fusion can make up for the limitations of a single mode and provide more comprehensive and accurate emotion recognition results. For example, when students write with their heads down, their facial expressions may not fully capture their emotional state. In this case, combining speech intonation and body movement information can enhance the accuracy and reliability of emotion recognition. Through the fusion processing of multimodal data, the system can better understand the true emotional state of students and provide rich emotional data for subsequent result analysis.

In addition, the accuracy of facial expression recognition also depends on the personalized modeling of different individuals. Due to individual differences in facial features and expressions among students, a unified recognition model may not be able to adapt to all individuals. Therefore, introducing personalized modeling mechanisms into facial expression recognition systems and establishing personalized facial expression recognition models for each student can significantly improve recognition accuracy. This process typically involves learning and adapting individual features, dynamically updating the model using previously collected data to continuously optimize recognition performance.

The final output of the facial expression recognition process is a detailed record of the emotional state of each student throughout the entire teaching process. These records include time series data of emotional states as well as emotional fluctuations in different teaching stages. By organizing and analyzing these emotional data, comprehensive emotional feedback can be provided, laying a solid foundation for subsequent result analysis and teaching feedback. The facial expression recognition stage is not only the initial processing stage of emotional data but also the core stage of the entire auxiliary teaching and student evaluation method. Its accuracy and effectiveness directly affect the final quality of teaching feedback.

### Result Analysis

In the medical education–assisted teaching and student evaluation method based on facial expression recognition, the result analysis stage plays a bridging role, which is a key step in transforming the identified emotional data into a deep understanding of teaching effectiveness and student learning status. The result analysis first involves the organization and preprocessing of emotional data, including smoothing of time series, detection and removal of outliers, and statistical analysis of the distribution of different emotional categories. Through preliminary processing of emotional data, the emotional change curves of each student at different time periods can be obtained, which provides a foundation for further in-depth analysis.

Based on the organized emotional data, statistical analysis and data mining techniques are further used to reveal the relationship between students’ emotional states and teaching activities. Specifically, through regression analysis, correlation analysis, and other methods, the impact of different teaching stages, content, and methods on students’ emotional states can be explored. For example, analyzing the changes in students’ concentration, confusion, and participation in different teaching stages, such as teacher lectures, interactive questioning, and experimental operations, in order to evaluate the teaching effectiveness of these stages. In addition, through cluster analysis and pattern recognition, student groups with similar emotional change patterns can be identified, providing a basis for personalized teaching and hierarchical guidance.

To gain a deeper understanding of the teaching significance behind students’ emotional data, result analysis should also be combined with the specific context of classroom teaching and the teacher’s teaching design. By synchronously analyzing emotional data and teaching videos, it is possible to identify which specific teaching behaviors have caused students’ emotional fluctuations. For example, when students exhibit general confusion or anxiety at a certain point in time, it is possible to trace back to the teaching content and the teacher’s explanation during that period and identify the difficult issues or unclear explanations that may have caused confusion for students. This situational analysis not only helps diagnose problems in teaching but also provides specific reference suggestions for teachers to improve their teaching.

In the result analysis stage, special attention should be paid to the handling of individual differences. Due to significant differences in emotional performance and learning styles among each student, a unified analysis method may not fully reflect an individual’s learning status. Therefore, the result analysis should be combined with personalized emotional data for targeted analysis. For example, by longitudinal tracking and comparative analysis of a student’s emotional change data, it is possible to evaluate their emotional response under different courses and teaching methods and identify the most suitable teaching strategy for their learning style. In this way, not only can it improve the accurate assessment of individual learning status, but it can also provide a scientific basis for personalized teaching.

Finally, the output of the result analysis stage is a comprehensive evaluation report of the entire teaching process and students’ emotional state. These reports include statistical results of emotional data, trend analysis of emotional changes, emotional feedback in teaching processes, and personalized teaching recommendations. Through these detailed analysis reports, teachers can comprehensively understand the teaching effectiveness and students’ learning status, adjust teaching strategies and methods in a targeted manner, and thus achieve continuous improvement in teaching quality. Result analysis is not only a simple processing of emotional data but also a data-driven teaching improvement process, and its accuracy and scientificity directly affect the effectiveness and operability of teaching feedback.

### Teaching Feedback

In the medical education–assisted teaching and student evaluation method based on facial expression recognition, the teaching feedback link is the terminal link of the entire process, aiming to transform the conclusions drawn from the result analysis into specific teaching improvement measures. Teaching feedback is not only feedback on students’ learning status and teaching effectiveness but also guidance for future teaching design and strategies. Medical education has its unique characteristics, including highly specialized knowledge content, rigorous practical skills training, and comprehensive requirements for students’ overall quality, which makes the teaching feedback link particularly important in medical education.

First, in the teaching feedback stage, the emotional data and behavioral patterns identified in the result analysis need to be fed back to the teacher. This process not only includes the aggregation and presentation of emotional data but also requires providing detailed explanations and analysis reports in conjunction with specific teaching contexts and classroom activities. For example, in the result analysis, it is found that some students exhibit obvious confusion or anxiety in specific teaching stages. Teaching feedback should provide a detailed explanation of the possible reasons for these emotional changes and provide corresponding teaching improvement suggestions. For medical education, this meticulous feedback helps teachers to explain complex medical concepts or skill operations in a more targeted manner in subsequent teaching, improving teaching effectiveness.

Second, the teaching feedback process should also focus on providing individual feedback and guidance to students. Students in medical education not only need to master a large amount of theoretical knowledge but also need to possess solid clinical skills and good professional ethics. Personalized feedback can help students understand their weak areas in learning and provide specific improvement suggestions. For example, through facial expression recognition and emotion analysis of students in simulated clinical operations, feedback can point out the possible tension or lack of confidence that students may have during the operation process and suggest that they improve their operational skills and psychological qualities through more practice or psychological counseling. Such personalized feedback not only helps students make progress in their studies but also assists them in better coping with various challenges in their future careers.

In addition, the teaching feedback process should also take into account the characteristics of team learning and collaborative practice in medical education. Medical education not only focuses on individual learning but also emphasizes the importance of teamwork and collective decision-making. Therefore, teaching feedback should include emotional and behavioral analysis of student teamwork, identifying potential communication barriers or collaboration issues within the team. For example, through facial expression recognition technology, analyzing students’ emotional states during team discussions or collaborative tasks can identify which members may have issues with insufficient participation or poor communication and provide suggestions for improving the team’s collaboration methods. In this way, not only can it improve the efficiency of team learning, but it can also cultivate students’ cooperation ability and team spirit, which are key qualities in medical education.

Finally, the teaching feedback process should focus on long-term effectiveness evaluation and continuous improvement. Medical education is a long-term process, and the cultivation of students’ learning outcomes and professional qualities requires continuous attention and improvement. Through regular emotional data analysis and teaching feedback, teachers can dynamically adjust teaching strategies and continuously optimize teaching content and methods. For example, regularly reviewing and summarizing students’ emotional data and learning performance, evaluating the effectiveness of teaching improvement measures, and proposing new teaching improvement plans based on this. In this way, teaching feedback is not only a reflection and improvement of current teaching activities but also a continuous and dynamic process of improving teaching quality.

In summary, the feedback loop in teaching is of great significance in medical education–assisted teaching and student evaluation methods based on facial expression recognition. Through meticulous analysis of emotional data and personalized feedback, not only can teaching effectiveness be improved and students overcome learning difficulties, but also team collaboration and long-term improvement of teaching quality can be promoted. The particularity of medical education requires that the teaching feedback process pays more attention to individual differences, teamwork, and continuous improvement in order to comprehensively enhance the quality of medical education and the comprehensive quality of students.

## Advantages and Limitations of the Method

### Advantages of Methods

The medical education–assisted teaching and student evaluation methods based on facial expression recognition have multiple advantages, which are particularly significant in the special context of medical education. First, through real-time facial expression recognition technology, it is possible to objectively and instantly capture and analyze students’ emotional states in the classroom. This real-time data acquisition and analysis enables teachers to timely understand students’ emotional changes during the teaching process, especially their understanding of complex or abstract medical concepts. When students show negative emotions, such as confusion or anxiety, teachers can immediately adjust teaching strategies, such as providing more detailed explanations or adding interactive elements, to alleviate students’ emotions and improve teaching effectiveness. This real-time feedback mechanism significantly enhances the dynamic adjustment ability of classroom teaching, ensuring that every student can keep up with the teaching progress.

Second, methods based on facial expression recognition can provide refined personalized teaching feedback. The content of medical education is complex and covers a wide range of professional knowledge, and students’ understanding and mastery vary greatly. By analyzing students’ facial expression data in different teaching stages, specific difficulties and emotional fluctuations of individuals in the learning process can be accurately identified. For example, when studying anatomy, if a student shows persistent confusion in a specific chapter, the facial expression recognition system can capture and record this phenomenon and provide targeted learning suggestions for the student in teaching feedback. This personalized feedback mechanism can not only help students overcome obstacles in learning but also improve their learning efficiency and effectiveness, thereby achieving the goal of teaching according to their aptitude.

In addition, this method has unique advantages in cultivating students’ clinical practice skills and comprehensive qualities. Medical education not only emphasizes the mastery of theoretical knowledge but also pays more attention to the training of clinical skills and the cultivation of professional qualities. Through facial expression recognition technology, students’ emotional states can be monitored and analyzed in real time during simulated clinical operations and actual clinical training. For example, during simulated surgical procedures, the system can capture students’ emotional changes, such as tension, anxiety, or confidence, and use these data to evaluate their proficiency and psychological resilience. This evaluation method is more objective and comprehensive than traditional manual observation, which helps teachers to strengthen students’ practical training and psychological development in teaching feedback and improve their clinical and emergency response abilities.

In addition, the evaluation method based on facial expression recognition has also demonstrated significant advantages in cultivating teamwork and communication skills. Many tasks and projects in medical education require students to collaborate as a team, and effective communication and collaboration among team members are key to success. Facial expression recognition technology can monitor and analyze the emotional states of team members during collaboration, identifying potential communication barriers or collaboration issues. For example, in team discussions or collaborative tasks, if the system detects insufficient participation or low mood among certain members, it can promptly provide feedback to the teacher or team leader, prompting them to take measures to improve the team’s collaborative atmosphere. This team evaluation and feedback based on emotional data not only improves the efficiency of team learning but also cultivates students’ communication skills and teamwork spirit.

Finally, the teaching evaluation method based on facial expression recognition has the advantage of continuous improvement. Medical education is a long-term and dynamic process that requires continuous adjustment and optimization of teaching strategies. By regularly collecting and analyzing emotional data, teachers can continuously evaluate teaching effectiveness and identify and improve deficiencies in teaching. For example, by tracking students’ emotional changes and learning performance over the long term, evaluating the effectiveness of different teaching methods and content, and continuously optimizing and adjusting them in subsequent teaching. This data-driven continuous improvement mechanism ensures the high quality of medical education and the efficiency of student learning, meeting the needs of modern medical education for refined and personalized teaching.

### Limitations of the Method

Although facial expression recognition–based methods for medical education auxiliary teaching and student evaluation offer significant advantages, such as real-time feedback and objective assessment, their limitations, including issues related to data privacy, cross-cultural recognition accuracy, and the complexity of emotional interpretation, cannot be overlooked. First, the accuracy and reliability of facial expression recognition technology face challenges in practical applications. Facial expressions are influenced by various factors, including lighting, shooting angle, and facial occlusion, which may result in inaccurate recognition results. In addition, there are significant differences in facial expressions and emotional expression styles among individuals, which limits the applicability of universal expression recognition models in personalized applications. Especially in medical education, students may have more subtle emotional expressions when faced with complex professional content, which further increases the difficulty of facial expression recognition.

Second, the dependency of facial expression recognition technology is an important issue. This technology relies on high-quality video data and advanced computing devices, and in practical teaching environments, especially with limited resources, it is difficult to ensure that every classroom is equipped with sufficient cameras and computing power. This not only increases implementation costs but may also lead to incomplete or low-quality data collection, thereby affecting the accuracy and reliability of evaluation results. In addition, prolonged video surveillance may cause students’ resistance and affect their natural performance, thereby interfering with the authenticity of emotional data.

Furthermore, facial expression recognition technology mainly focuses on facial expressions, and emotions and psychological states are complex and multidimensional. Relying solely on facial expression data is difficult to fully reflect students’ true emotions and learning status. In medical education, students’ stress, anxiety, and cognitive load are often complex and varied, and these internal states may not be fully expressed through facial expressions. Therefore, relying solely on facial expression recognition for emotional assessment has limitations and may lead to a one-sided understanding of students’ learning status. In addition, the interpretation of emotional data is also subjective, and different teachers may have different interpretations of the same emotional data, which affects the consistency and effectiveness of teaching feedback.

In addition, privacy protection and ethical issues are major challenges based on facial expression recognition technology. Collecting and analyzing students’ facial expression data involve personal privacy and must strictly comply with relevant laws, regulations, and ethical guidelines. In practical operation, how to ensure the security, anonymity, and transparency of data use is a complex and sensitive issue. If not handled properly, it may cause concerns for students and parents and even lead to legal disputes. In addition, long-term monitoring and data analysis may bring psychological pressure, affecting students’ learning experience and mental health.

Finally, there is uncertainty in the application effectiveness of facial expression recognition–based evaluation methods in actual teaching improvement. Although emotional data can provide some reference, improving teaching effectiveness involves multiple factors, including teaching content, teaching methods, and teacher quality. Relying solely on emotional data is difficult to comprehensively improve teaching quality. Therefore, in the application process, it is necessary to combine other evaluation methods and means and comprehensively analyze and improve teaching strategies to ensure the comprehensiveness and scientificity of teaching feedback.

## Conclusions and Prospect

In this paper, we explore the application of facial expression recognition technology in medical education and its potential in assisting teaching and student assessment. Through in-depth research and practical application analysis of facial expression recognition technology, we have found that this technology can significantly enhance teacher-student interaction, improve teaching effectiveness, and provide strong support for personalized education. Specifically, facial expression recognition technology can capture and analyze students’ facial expressions in real time, helping teachers understand students’ emotional states and understanding in a timely manner, thereby adjusting teaching strategies and providing more targeted guidance.

In addition, facial expression recognition technology can also serve as an effective student assessment tool, providing detailed feedback data for teachers by quantitatively analyzing students’ emotional reactions and participation in the classroom. These data can not only be used to evaluate students’ learning outcomes but also to improve curriculum design and teaching methods, thereby achieving a comprehensive improvement in educational quality.

Looking ahead to the future, with the continuous development and improvement of facial expression recognition technology, its application prospects in medical education will be even broader. We foresee that future medical education will become more intelligent and personalized, and facial expression recognition technology will be combined with other advanced technologies such as artificial intelligence and big data analysis to jointly build a comprehensive and dynamic education ecosystem. At the same time, with the popularization of technology and the accumulation of application experience, we also need to pay attention and solve the ethical and privacy issues that technology may face in practical applications, ensuring the sustainable development of technology in the field of education.

In short, facial expression recognition technology, as an innovative tool, can not only improve the teaching effectiveness and evaluation efficiency of medical education but also provide new ideas and directions for the future development of medical education. We believe that with the deepening of research and the promotion of application, facial expression recognition technology will bring more innovation and change to medical education and help cultivate more outstanding medical talents.

## Abbreviations

CNN: convolutional neural network
SVM: support vector machine
