# Correction: Auxiliary Teaching and Student Evaluation Methods Based on Facial Expression Recognition in Medical Education

**DOI:** 10.2196/89669

**Published:** 2026-01-14

**Authors:** Xueling Zhu, Roben A Juanatas

**Affiliations:** 1 College of Computing and Information Technologies, National University, 551 Mariano Fortunato Jhocson Street, Sampaloc, Manila, 1008, Philippines, 86 13966689261; 2 College of Big Data and Artificial Intelligence, Anhui Xinhua University, Hefei, Anhui, China

In “Auxiliary Teaching and Student Evaluation Methods Based on Facial Expression Recognition in Medical Education” [1], the authors noted one error in the author affiliation list.

A second affiliation has now been added for author XZ, as follows:

College of Big Data and Artificial Intelligence, Anhui Xinhua University, Hefei, Anhui, China

The correction will appear in the online version of the paper on the JMIR Publications website, together with the publication of this correction notice. Because this was made after submission to PubMed, PubMed Central, and other full-text repositories, the corrected article has also been resubmitted to those repositories.
